# Plant Protein but Not Animal Protein Consumption Is Associated with Frailty through Plasma Metabolites

**DOI:** 10.3390/nu15194193

**Published:** 2023-09-28

**Authors:** Toshiko Tanaka, Jayanta K. Das, Yichen Jin, Qu Tian, Ruin Moaddel, Ann Zenobia Moore, Katherine L. Tucker, Sameera A. Talegawkar, Luigi Ferrucci

**Affiliations:** 1 Longitudinal Studies Section, Translational Gerontology Branch, National Institute on Aging, Baltimore, MD 21224, USAferruccilu@grc.nia.nih.gov (L.F.); 2 Department of Exercise and Nutrition Sciences, Milken Institute School of Public Health, The George Washington University, Washington, DC 20052, USA; 3 Laboratory of Clinical Investigation, National Institute on Aging, Intramural Research Program, NIH, Baltimore, MD 21224, USA; 4 Department of Biomedical and Nutrition Sciences, University of Massachusetts Lowell, Lowell, MA 01854, USA

**Keywords:** protein consumption, frailty index, aging, epidemiology

## Abstract

There is evidence that the association of protein intake and frailty may depend on the source of dietary protein. The mechanism underlying this association is not clear. In this study, we explore circulating metabolites as mediators of the relationship between dietary protein and of frailty in participants of the Baltimore Longitudinal Study of Aging (BLSA). Cross-sectional analyses in 735 BLSA participants of associations between plant and animal protein intake and frailty. Usual protein intake from plant and animal sources were estimated with a Food Frequency Questionnaire (FFQ) and frailty was assessed with a 44-item Frailty Index (FI). Compared with the lowest quartile, higher quartiles of plant, but not animal, protein were associated with lower FI. Twenty-five plasma metabolites were associated with plant protein intake; of these, fifteen, including phosphatidylcholines, cholesterol esters, sphingomyelins, and indole metabolites, mediated the association between plant protein intake and FI. The protective association between plant protein consumption and FI is mediated by lower abundance of lipid metabolites and higher abundance of tryptophan-related metabolites.

## 1. Introduction

Adults 65 years of age and older comprise a rapidly growing segment of the population worldwide [1]. With older age, there is an increased burden of disease and higher prevalence of mobility difficulties. Frailty is a geriatric syndrome characterized by vulnerability to external or internal stressors due to decline across multiple physiologic systems. The presence of frailty or being on the higher spectrum of frailty has been associated with poorer health outcomes, including increased risk of falls, and early death [2].

With the growing number of people living into their golden years, there is an increasing need to develop new approaches to delay or prevent the onset or progression of frailty. In particular, identification of behavioral modifications that promote healthy aging that will have a significant public health impact. In this context, various aspects of diet quality that may promote healthy longevity have been investigated. Adherence to healthy dietary patterns, such as the Mediterranean diet, has been associated with reduced risk of having or developing frailty [3,4]. In addition to dietary pattern, the macronutrient composition of the diet, and the quality of each macronutrient have been shown to have important health consequences. Several studies have shown that the quality of carbohydrate and fat intakes were associated with frailty [5,6]. Protein is a particularly important macronutrient for older adults, and some researchers argue that they need more protein than what is currently recommended for health maintenance [7]. In addition to protein quantity, there is evidence that the source of protein—from plant including vegetable, legume, and nuts, or animal such as dairy and meats—could be an important factor in relation to frailty [8,9]. While there are conflicting results, some studies have shown that the association of protein intake with frailty differs based on the source of protein, where plant may be a more favorable source to reduce the risk or severity of frailty [9,10]. The mechanisms underlying the association between plant or animal protein intake and frailty have not yet been explored. To address this question, we examined cross-sectional associations of plant and animal protein intake with the frailty index, and further explored plasma metabolites as possible mediators of these relationships in participants of the Baltimore Longitudinal Study of Aging (BLSA).

## 2. Materials and Methods

### 2.1. Study Design and Participants

The BLSA is a rolling enrollment cohort study of aging that started in 1958. Detailed protocols for the BLSA study have been provided elsewhere [11]. Briefly, BLSA participants are individuals primarily residing in the Washington DC-Baltimore area. At the time of enrollment, participants must be free of most chronic diseases (except controlled hypertension), mobility and cognitive disability. Once enrolled, participants are followed at different time intervals, based on their age (every 4 years if <60 years; every 2 years if 60–80 years, and annually if >80 years of age). A wide range of data are collected during a scheduled 3-day visits at the Clinical Research Unit of the Intramural Research Program of the National Institute on Aging (IRP, NIA). For participants that are not able to travel to the clinical center, data are collected through a home visit. This analysis was conducted on 735 participants over 65 years of age, the most recent visit with data on dietary intake and frailty. Each participant provided informed consent at each visit. The BLSA study protocol (Protocol number 03-AG-0325) was approved by the National Institutes of Health Intramural Research Program Institutional Review Board.

### 2.2. Dietary Assessment

From 2005, habitual daily consumption of dietary protein was assessed was captured using a Food Frequency Questionnaire (FFQ) that has been validated against data derived with diet diary [12]. The FFQ was implemented by trained BLSA staff using paper questionnaires and computer-based REDCap surveys beginning in 2016. Nutrient and energy composition was generated using the University of Minnesota Nutrient Data System for Research (NDS-R) program. Participants that reported improbable daily energy intake (<600 or >4800 kcal) were excluded from the analysis. Protein intake was expressed as percent of energy from total protein, animal protein, or plant protein, calculated by multiplying the grams of individual protein intake by 4 kcal/g and dividing by total energy intake. Protein consumption was then categorized into quartiles and assigned quartile-specific median values and analyzed as categorical or continuous variables.

The Mediterranean style diet score (MDS) was used as a measure overall diet quality. The MDS reflects a cumulative score of the consumption of nine food groups and nutrients [13]. These included food components that are considered to be beneficial (vegetables, legumes, fruits and nuts, whole grain, fish, monounsaturated-to-saturated fatty acid ratio), or detrimental (meat, dairy, as well as alcohol). For the beneficial foods, consumption above the median value was given a score of 1, while consumption below the median of detrimental foods was given a score of 1. For alcohol consumption, moderate intake was given a 1 while high or low intake did not contribute to the score. The total MDS ranged from 0 to 9, where higher score indicated better adherence to a Mediterranean style diet.

### 2.3. Plasma Metabolomic Assessment

Overnight fasted plasma samples were obtained and stored at −80C until used for metabolomic assessment. All metabolomic assessments were performed at Biocrates Life Sciences AG (Innsbruck, Austria). Plasma metabolites were assessed using liquid chromatography-tandem mass spectrometry (LC–MS/MS) at using the MxP^®^ Quant 500 kit using standard procedures. Flow injection analysis-tandem mass spectrometry (FIA-MS/MS) using a 5500 QTRAP^®^ instrument (AB Sciex, Darmstadt, Germany) with an electrospray ionization source was used to analyzed lipids and hexose. The same 5500 QTRAP^®^ instrument was used to assess additional twenty-six biochemical classes (amino acids, related amino acids, carboxylic acids, fatty acids, indole derivatives, biogenic amines, bile acids, cresols, alkaloids, amine oxides, hormones, vitamins and cofactors, and nucleobases related metabolites), and measured by LC–MS/MS. Quantification of metabolites was performed using MS software (Sciex Analyst^®^ version 1.6) and processed with MetIDQ™ software version “Oxygen” (Biocrates). Using internal calibrators and predefined tolerance thresholds, all analytes were deteremined to have measurement accurancies within normal range. Further quality control was conducted by removing metabolites with >30% of observations below the limit of detection (LOD; n = 156). For the remaining 466 metabolites, those values below LOD were set to half the minimum concentrations. Each metabolite was log2 transformed and standardized (mean 0, standard deviation = 1) before analysis.

### 2.4. Construction of Frailty Index (FI)

A 44-item frailty index (FI) was constructed following the procedures outlined by Searle et al. [14] and has been previously described for the BLSA [5]. For the BLSA, the deficit items that were used for the index were: Self-reported difficulties conducting activities (instrumental) of daily living (ADL/IADL), overall self-rated health assessed using standard questionnaire [15], measure of depressive symptoms using the Center for Epidemiologic Studies Depression (CES-D) scale [16], measure of cognitive function as assessed with the Mini-Mental State Examination [17], prevalence of 14 common chronic aging conditions, unintentional weight loss of 5% over a 1 year period, sedentary behavior (lowest quartile of combination of outdoor work, heavy chores, brisk walking, high intensity and moderate exercise), slow walking speed (sex and height), low grip strength (stratified by sex and BMI). The final FI represented the sum of the 44 items in participants with at least 80% of complete data at the study visit.

### 2.5. Assessment of Covariates

Demographic and anthropometric parameters, such as age, sex, and years of education were collected through a structured interview and medical examination. Body mass index (BMI) reflects weight (kg) divided by height squared (m^2^).

### 2.6. Statistical Analysis

Differences in continuous and categorical variables by quartiles of protein consumption were assessed using one-way ANOVA and chi-square tests, respectively. Associations between quartiles of plant and animal protein and FI were conducted using multiple linear regression, where quartile of protein intake was the main exposure and FI the independent variable. Both quartiles of plant and animal protein were included in the same regression model. The regression models were further adjusted for age, sex, BMI, self-reported race, smoking status, calendar year of BLSA visit, total energy intake (kcal/day), % energy from total fat, and alcohol intake (g/day). A sensitivity analysis that accounted for overall diet quality was conducted by including the MDS as an additional covariate.

Associations between quartiles of plant protein intake and metabolites were assessed with multiple linear regression. For each metabolite, the plant protein intake was included in the model using the quartiles of respective protein intake as a linear main exposure and abundance of metabolite the dependent variable. The analyses included all the covariates included in the first analysis of plant protein intake and FI. To account for multiple comparison of 466 metabolites, a false discovery rate (FDR) correction was applied and FDR-corrected *p* ≤ 0.05 was considered significant.

Finally, to evaluate the potential mediating effect of metabolites in the association of plant protein intake and FI, causal mediation analysis was conducted using the *mediation* package in R version 3.6.3. In this analysis, quartile of plant protein intake (as a continuous variable) was set as the main exposure of interest, FI as the outcome, and individual plasma metabolite as the mediator. The direct effect reflects the association of plant protein on FI and the indirect effect measures the association of plant protein intake on FI through the metabolomic mediator. The proportion of mediation expressed as a percentage reflects the degree of mediating effect. For these analyses, significant indirect effect at *p* ≤ 0.05 was considered as significant mediation.

For all statistical analyses, the R Statistical Software version 3.6.3 was used.

## 3. Results

### 3.1. Cross-Sectional Associations of Protein Intake with Frailty Index

In analytical sample consisted of men and women over 65 years of age (mean age 77 ± 7.5 years old). The mean percentage of total energy from protein was 17.7%, with greater proportion of total protein intake from animal sources (67%), than plant. The basic characteristics of the BLSA participants by quartiles of plant and animal protein are displayed in Table 1. Being in the higher quartiles of animal protein intake was associated with higher BMI and higher proportion of energy from fat, and with lower MDS score, total energy intake, % energy from carbohydrate, % plant protein, and alcohol consumption. Being in the higher quartiles of plant protein intake was associated with lower prevalence of smoking, BMI, % energy from fat, % energy from animal protein, alcohol consumption, and higher MDS score, and % energy from carbohydrates (Table 1). Plant protein consumption was also associated with self-reported race, with a lower proportion of White, and higher proportion of “other” race in the higher quartiles plant protein intake.

Consumption of plant protein (as % energy) was negatively associated with FI, while animal protein and total protein intake were not associated with FI (Table 2 and Table S1). An increase in quartile of protein intake was associated with an 0.008 unit decrease in FI, this association was independent of total energy, total fat, alcohol intake, age, sex, self-reported race, smoking, year of visit, and BMI. The association remained significant after further adjustment for MDS. In model 1, a significant difference was observed between the lowest quartile of vegetable protein intake and the highest quartile (*p* = 0.003). This association was attenuated, but remained significant, after adjusting for MDS. When treating protein intake as a categorical variable, significant differences were observed for the comparison of the highest quartile of protein intake compared to the lowest quartile. This association was attenuated after adjustment for overall diet quality.

### 3.2. Plasma Metabolomic Profile of Vegetable Protein Intake

**Figure 1:**
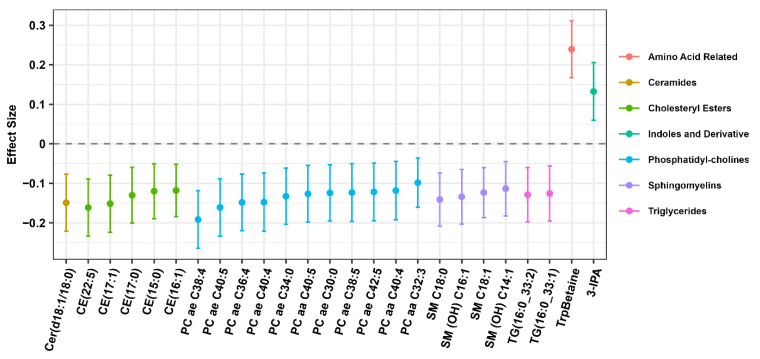
Association of plasma metabolites with quartile of plant protein intake. This dotplot displays the effect size or beta coefficient from the linear regression model predicting metabolite abundance with quartiles of plant protein intake.

Of the 466 plasma metabolites assessed, 25 were associated with quartiles of plant protein consumption after adjustment for total energy, total fat, alcohol intake, age, sex, self-reported race, smoking, year of visit, and BMI (Figure 1, Table S2). Higher consumption of plant protein was associated with lower abundances of various lipid metabolites including ceramides (1), cholesterol esters (5), phosphatidylcholines (11), sphingomyelins (4), and triglycerides (2). Conversely, higher consumption of plan protein was associated with higher abundance of tryptophan betaine (TrpBetaine) and indole-3-propionic acid (3IPA). After adjustment for overall diet quality or MDS, TrpBetaine, phosphatidylcholine ae C40:5 (PC(o-20:1(11Z)/20:4(8Z,11Z,14Z,17Z)), Cholesteryl ester 17:1, and Phosphatidylcholine ae C42:5 (PC(o-22:1(13Z)/20:4(8Z,11Z,14Z,17Z)) remained significantly associated with plant protein intake (Table S2).

### 3.3. Plasma Metabolites Mediate the Association between Plant Protein Intake and Frailty Index

Of the 25 metabolites associated with plant protein consumption, 15 partially mediated the associations between plant protein consumption and FI (Table 3). These included cholesterol esters (4), sphingomyelins (4), phosphatidyl-cholines (6), and 3IPA. The percent mediation ranged from 8.5% to 16.6%.

## 4. Discussion

In this cross-sectional analysis of older men and women of the BLSA, we find that higher habitual consumption of plant protein is negatively associated with FI while animal and total protein consumption was not associated with FI. The protective association observed was independent of overall dietary quality, as assessed by the Mediterranean diet score. The plasma metabolomic signature of vegetable protein consumption indicated an overall depletion of lipid species, including phosphotidylcholines, sphyngomyelins, cholesterol esters, ceramides and triglycerides, and greater abundance of tryptophan-related metabolites. A subset of the lipid and tryptophan-related metabolites mediated the association of vegetable protein consumption and frailty.

The importance of dietary protein in aging has been an active area of research for many years. With the observed decline in muscle mass and strength with aging, results from studies that support the benefits of protein intake have led some experts to suggest that the recommended daily allowance of protein in older individuals be increased from 0.8 g/kg/d to 1.2 g/kg/d, or greater, of high quality protein to maintain optimal health, particularly in older persons with a mild pro-inflammatory state [7,18]. Greater protein consumption has been linked with reduced prevalence and incidence of frailty in many, but not all studies [19,20,21,22,23]. In one of the largest studies of 24,417 participants of the Women’s Health Initiative (WHI), higher quantiles of protein intake were inversely associated with incidence of frailty over a 3-year follow-up period [21]. Interestingly, in this study, protein intake assessed by FFQ was calibrated using urinary nitrogen to objectively assess protein intake over a 24 hr period. The association between protein intake and frailty was stronger when calibrated protein intake was used. Like the WHI study, other studies support the beneficial effects of protein consumption on frailty; however, not all studies show significant associations. For example, in the MrOS study of 5922 older community-dwelling men, there was no significant association between quantiles of protein intake with prevalence of frailty, defined using the Fried frailty phenotype definition, at baseline. In the same study, among those who were robust at baseline, there were no significant associations between baseline protein consumption and incidence of frailty during the ~4.6 year follow up period [23].

There has been great interest in understanding whether quantity or quality of protein consumption could have important implications on risk of frailty. Some studies show differential associations with frailty depending on the source of dietary protein. A recent study of 85,871 female participants of the Nurses’ Health study, plant protein intake was inversely associated with risk of developing frailty over a 22-year follow up period [9]. Conversely, consumption of animal protein was associated with increased risk of developing frailty. Further, replacing animal protein with plant protein was associated with 38% reduced risk of frailty. In the same study, consumption of red meat, particularly processed red meat, was significantly associated with increased risk of frailty [10]. In the BLSA, the majority of dietary protein was from animal sources, consistent with other cohort studies [24]. We found that plant protein was inversely associated with FI in a dose-dependent manner, while animal protein was not associated with FI. Plant protein consumption was significantly associated with MDS score, suggesting that participants who consume more plant proteins have greater adherence to a healthier dietary pattern. Therefore, it is possible that the association between plant protein is not due to protein per se, but, at least in part, due to healthy patterns of eating. However, when adjusting for overall diet quality, plant protein consumption remained significantly associated with FI.

The most significant plasma metabolite associated with plant protein intake was tryptophan betaine (TrpBetaine). TrpBetaine or hypaphorine, is an indolic compound of L-tryptophan with three methyl groups. This metabolite is found in legumes, such as lentils, peanuts, and chickpeas [25]. We have previously reported that greater abundance of TrpBetaine was associated with greater adherence to healthy dietary patterns, including the MDS [26,27]. An intervention using the DASH dietary pattern reported higher TrpBetaine following the intervention period [28]. The OmniHeart Trial is a randomized crossover feeding study that tested three diets with differing macronutrient contents in 156 participants [29]. In this study, TrpBetaine increased following a protein-rich diet, where half of the proteins were plant proteins. Together these data, along with our results, support TrpBetaine as a marker of plant protein consumption in humans. In the mediation analysis, the mediating effect of TrpBetaine on the relationship between plant protein and FI was not significant. However, TrpBetaine has been shown to have health promoting anti-inflammatory effects in response to infection, thus it is plausible that plant protein consumption can reduce inflammation through increases in TrpBetaine [30,31].

Tryptophan can be metabolized by the gut mircriobiota to the indolic compounds: indoxyl sulfate, 3-IPA and/or indole-3-aldehyde (IAAA). While IAAA was not associated with plant protein intake, 3-IPA was a metabolite found in greater abundance with plant protein intake, which significantly mediates associations with FI. Conversely, while not significant indoxyl sulfate had a trending (*p* = 0.097) negative abundance with plant protein intake. Indoxyl sulphate (IS), a uremic toxin that is produced in the gut with pro-inflammatory effects, conversely 3-IPA has been shown to anti-inflammatory effects. Consistent with what was observed in the BLSA, previous observational studies reported higher 3-IPA abundance with greater adherence to plant-based dietary patterns [32]. Increased serum 3-IPA was observed in an 8-week trial of polyphenol-rich diet [33]. Increases in 3-IPA can reduce risk of frailty through their anti-oxidant properties [34,35] and regulation of immune function, via Aryl hydrocarbon receptor activation [36,37]. Observational studies have shown that circulating 3-IPA was associated with reduced risk of diabetes [38] and cardiovascular disease [39]. Taken together, these studies and our results support 3-IPA as a metabolite linked with better age-related health outcomes.

Greater plant protein consumption was associated with lower lipid metabolites, including ceramides, cholesterol esters, phosphatidyl-cholines, sphingomyelins, and triglycerides. We have previously reported depletion of the same lipid metabolites in association with adherence to healthy dietary patterns [26]. Further, the DASH diet intervention study [40] and OmniHeart intervention study [29] showed lower abundance of these lipid species following a DASH or protein rich diet, respectively. Our study suggests that these lipids may also be informative markers of plant protein intake. We further show that cholesterol esters, sphingomyelins, and phosphatidylcholines mediated the association between plant protein and frailty. This is consistent with prior studies, which have reported higher abundance of these lipid metabolites associated with physical frailty [41].

The strength of the current study include that the analysis was conducted in a deeply phenotyped cohort with a well-established definition of frailty, using the deficit accumulation model. The dietary assessment was conducted using a validated FFQ, and the analytical model considered various aspects of diet that could confound the relationship between plant protein and frailty. There are also important limitations of the study that need to be discussed. First, the study design is cross-sectional, thus no causal relationships between plant protein consumption and metabolites or FI can be established. While the dietary assessment instrument has been validated and designed to assess habitual dietary intake, self-reported dietary data are prone to biases and reporting errors. Further, plasma metabolites may be more sensitive to acute diet rather than habitual diet. However, our results are supported by the body of previous literature from observational and intervention studies that suggest these metabolites as potential biomarkers of protein or dietary patterns with higher plant protein intake. The targeted metabolomic platform has good coverage of plasma metabolites, but there are likely other metabolites linked with plant protein consumption that were not captured in this study. It is important to complement our findings with metabolomic analysis using other methodologies, including untargeted metabolomic studies. Finally, this study was conducted in BLSA participants who represent older individuals from higher socioeconomic demographic groups, thus generalizability to the general population or younger adults should be explored in future studies.

## 5. Conclusions

In this cross-sectional analysis of dietary protein source and FI, protein from plants, but not animal sources, were inversely associated with FI. Plant protein consumption is associated with lower abundance of various lipid species and increased abundance of tryptophan-related metabolites. These data suggest that diets rich in plant proteins may be an effective strategy to reduce the severity of frailty.

## Figures and Tables

**Table 1:** Demographic and dietary characteristics by quartile of animal or plant protein intake.

	Quartiles of %Animal Protein Intake (Median Value)					Quartiles of %Plant Protein Intake (Median Value)				
	Q1 (8%)	Q2 (11%)	Q3 (13%)	Q4 (16%)	*p*	Q1 (4%)	Q2 (5%)	Q3 (6%)	Q4 (7%)	*p*
n	184	184	184	183		184	184	184	183	
Age (y)	78.1 (7.40)	77.4 (7.34)	76.3 (7.14)	76.5 (7.83)	0.07	76.9 (7.82)	76.7 (7.27)	77.0 (7.17)	77.5 (7.56)	0.76
Sex (% male)	0.51 (0.50)	0.47 (0.50)	0.53 (0.50)	0.40 (0.49)	0.09	0.53 (0.50)	0.48 (0.50)	0.43 (0.50)	0.47 (0.50)	0.31
Self-reported Race					0.08					<0.001
White	128 (69.6)	137 (74.5)	131 (71.2)	134 (73.2)		137 (74.5)	136 (73.9)	141 (76.6)	116 (63.4)	
Black	40 (21.7)	34 (18.5)	47 (25.5)	44 (24.0)		44 (23.9)	43 (23.4)	37 (20.1)	41 (22.4)	
Other	16 (8.7)	13 (7.1)	6 (3.3)	5 (2.7)		3 (1.6)	5 (2.7)	6 (3.3)	26 (14.2)	
Smoking					0.74					0.01
Current (within 3 years)	4 (2.2)	4 (2.2)	6 (3.3)	4 (2.2)		7 (3.8)	7 (3.8)	2 (1.1)	2 (1.1)	
Former	78 (42.4)	69 (37.5)	81 (44.0)	83 (45.4)		93 (50.5)	80 (43.5)	71 (38.6)	67 (36.6)	
Non-smoker	102 (55.4)	111 (60.3)	97 (52.7)	96 (52.5)		84 (45.7)	97 (52.7)	111 (60.3)	114 (62.3)	
BMI (kg/m^2^)	26.7 (4.6)	26.2 (4.1)	28.1 (5.1)	28.0 (4.9)	<0.001	28.0 (5.3)	28.2 (4.7)	26.8 (4.5)	25.9 (4.1)	<0.001
Year of education	16.9 (2.6)	17.1 (2.9)	16.9 (2.6)	16.9 (2.5)	0.85	17.0 (2.5)	16.9 (2.7)	16.9 (2.6)	17.0 (2.8)	0.98
Mediterranean diet score	4.49 (1.62)	4.51 (1.58)	3.97 (1.72)	3.68 (1.61)	<0.001	3.30 (1.45)	3.85 (1.60)	4.33 (1.55)	5.17 (1.49)	<0.001
% Energy from carbohydrates	51.4 (8.5)	45.4 (6.4)	44.1 (6.48)	39.8 (7.1)	<0.001	40.5 (9.3)	43.7 (6.8)	47.1 (7.0)	49.6 (6.8)	<0.001
% Energy from fat	32.0 (7.1)	35.3 (5.3)	36.4 (5.90)	37.4 (6.4)	<0.001	36.9 (7.3)	36.6 (6.4)	34.8 (5.6)	32.7 (5.8)	<0.001
% Energy from protein	14.4 (2.1)	16.7 (1.48)	18.2 (1.2)	21.6 (2.8)	<0.001	17.6 (4.1)	18.0 (3.4)	17.5 (2.7)	17.8 (2.7)	0.57
% Energy from animal protein	7.97 (1.59)	10.8 (0.6)	12.9 (0.7)	16.7 (2.8)	<0.001	13.6 (4.0)	12.9 (3.4)	11.7 (2.8)	10.1 (3.0)	<0.001
% Energy from plant protein	6.45 (2.03)	5.84 (1.39)	5.28 (1.04)	4.93 (1.10)	<0.001	3.99 (0.59)	5.03 (0.19)	5.86 (0.29)	7.60 (1.47)	<0.001
Total energy (kcal/d)	1971 (846)	1945 (648)	1921 (780)	1694 (679)	<0.001	1943 (698)	1905 (776)	1836 (756)	1849 (765)	0.48
Alcohol intake (g/d)	14.9 (34.1)	12.8 (18.2)	8.21 (13.4)	6.05 (9.34)	<0.001	18.8 (33.5)	9.71 (17.6)	7.42 (11.9)	6.02 (11.8)	<0.001

Values represent mean (SD) or % (n).

**Table 2:** Association of animal and plant protein intake on frailty index.

	Model 1			Model 2		
	β	SE	*p*	β	SE	*p*
	Animal protein intake					
Per % increase	−0.001	0.001	0.327	−0.001	0.001	0.336
Q1	ref			ref		
Q2	−0.008	0.007	0.216	−0.007	0.007	0.275
Q3	−0.006	0.007	0.398	−0.006	0.007	0.409
Q4	−0.008	0.007	0.252	−0.008	0.007	0.272
	Plant protein intake					
Per % increase	−0.008	0.002	0.001	−0.006	0.003	0.034
Q1	ref			ref		
Q2	−0.004	0.007	0.576	−0.002	0.007	0.812
Q3	−0.013	0.007	0.053	−0.010	0.007	0.180
Q4	−0.023	0.008	0.003	−0.016	0.008	0.053

Model 1: association adjusted for age, sex, BMI, smoking, self-reported race, calendar year of BLSA visit, total energy intake (kcal/day), % energy from total fat, and alcohol intake (g/day). Model 2: Model 1 + Mediterranean diet score.

**Table 3:** Mediating effect of metabolite on plant protein association with frailty index.

	Average Direct Effect				Average Causal Mediated Effect				
Metabolite	β	LCI	UCI	*p*	β	LCI	UCI	*p*	% Mediation
3-IPA	−0.0068	−0.0108	−0.0018	0.004	−0.001	−0.0021	−0.0002	<0.001	12.89
CE(15:0)	−0.0087	−0.0137	−0.0044	<0.001	0.0009	0.0003	0.0018	<0.001	11.85
CE(17:0)	−0.0087	−0.0136	−0.0039	<0.001	0.001	0.0002	0.0019	0.004	12.72
CE(17:1)	−0.0089	−0.0133	−0.0042	<0.001	0.0011	0.0003	0.002	<0.001	14.36
CE(22:5)	−0.0090	−0.0137	−0.004	<0.001	0.0012	0.0004	0.0022	0.004	15.8
PC aa C32:3	−0.0084	−0.0126	−0.0037	<0.001	0.0007	0.0001	0.0015	0.036	8.54
PC ae C30:0	−0.0085	−0.0132	−0.0039	<0.001	0.0008	0.0002	0.0015	<0.001	9.77
PC ae C36:4	−0.0086	−0.0133	−0.0042	<0.001	0.0009	0.0002	0.0018	0.02	10.99
PC ae C38:5	−0.0084	−0.0126	−0.004	<0.001	0.0007	0.0001	0.0016	0.044	8.45
PC ae C40:5	−0.0087	−0.0128	−0.0043	<0.001	0.001	0.0001	0.0021	0.008	12.41
PC ae C42:5	−0.0084	−0.0126	−0.004	<0.001	0.0006	0.0001	0.0014	0.016	8.35
SM (OH) C14:1	−0.0088	−0.0134	−0.0044	<0.001	0.0011	0.0003	0.002	<0.001	13.85
SM (OH) C16:1	−0.0089	−0.0138	−0.0045	<0.001	0.0012	0.0004	0.0023	<0.001	15.27
SM C18:0	−0.0090	−0.0138	−0.0044	<0.001	0.0013	0.0005	0.0024	<0.001	16.61
SM C18:1	−0.0089	−0.0134	−0.0046	<0.001	0.0012	0.0004	0.0022	0.004	15.32

## Data Availability

Data from Baltimore Longitudinal Study of Aging are available through submission of research proposal through https://www.blsa.nih.gov/.

## Supplementary Materials

The following supporting information can be downloaded at: https://www.mdpi.com/article/10.3390/nu15194193/s1, Table S1: Association of total protein intake with frailty index; Table S2: Association of plasma metabolites with plant protein and animal protein.

## References

[1] Schumacher B.T., Di C., Bellettiere J., Lamonte M.J., Simonsick E.M., Parada H., Hooker S.P., Lacroix A.Z. (2023). Validation, Recalibration, and Predictive Accuracy of Published VO 2max Prediction Equations for Adults Ages 50–96 Yr. Med. Sci. Sports Exerc..

[2] Fried L.P., Tangen C.M., Walston J., Newman A.B., Hirsch C., Gottdiener J., Seeman T., Tracy R., Kop W.J., Burke G. (2001). Frailty in older adults: Evidence for a phenotype. J. Gerontol. A Biol. Sci. Med. Sci..

[3] Tanaka T., Talegawkar S.A., Jin Y., Bandinelli S., Ferrucci L. (2021). Association of Adherence to the Mediterranean-Style Diet with Lower Frailty Index in Older Adults. Nutrients.

[4] Kojima G., Avgerinou C., Iliffe S., Walters K. (2018). Adherence to Mediterranean Diet Reduces Incident Frailty Risk: Systematic Review and Meta-Analysis. J. Am. Geriatr. Soc..

[5] Tanaka T., Kafyra M., Jin Y., Chia C.W., Dedoussis G.V., Talegawkar S.A., Ferrucci L. (2022). Quality Specific Associations of Carbohydrate Consumption and Frailty Index. Nutrients.

[6] Chuy V., Gentreau M., Artero S., Berticat C., Rigalleau V., Peres K., Helmer C., Samieri C., Feart C. (2022). Simple Carbohydrate Intake and Higher Risk for Physical Frailty Over 15 Years in Community-Dwelling Older Adults. J. Gerontol. A Biol. Sci. Med. Sci..

[7] Phillips S.M., Chevalier S., Leidy H.J. (2016). Protein “requirements” beyond the RDA: Implications for optimizing health. Appl. Physiol. Nutr. Metab..

[8] Sandoval-Insausti H., Perez-Tasigchana R.F., Lopez-Garcia E., Garcia-Esquinas E., Rodriguez-Artalejo F., Guallar-Castillon P. (2016). Macronutrients Intake and Incident Frailty in Older Adults: A Prospective Cohort Study. J. Gerontol. A Biol. Sci. Med. Sci..

[9] Struijk E.A., Fung T.T., Rodriguez-Artalejo F., Bischoff-Ferrari H.A., Hu F.B., Willett W.C., Lopez-Garcia E. (2022). Protein intake and risk of frailty among older women in the Nurses’ Health Study. J. Cachexia Sarcopenia Muscle.

[10] Struijk E.A., Fung T.T., Sotos-Prieto M., Rodriguez-Artalejo F., Willett W.C., Hu F.B., Lopez-Garcia E. (2022). Red meat consumption and risk of frailty in older women. J. Cachexia Sarcopenia Muscle.

[11] Shock N.W., Greulick R.C., Andres R., Arenberg D., Costa P., Lakatta E., Tobin J.D. (1984). Normal Human Aging: The Baltimore Study of Aging.

[12] Talegawkar S.A., Tanaka T., Maras J.E., Ferrucci L., Tucker K.L. (2015). Validation of Nutrient Intake Estimates Derived Using a Semi-Quantitative FFQ against 3 Day Diet Records in the Baltimore Longitudinal Study of Aging. J. Nutr. Health Aging.

[13] Trichopoulou A., Costacou T., Bamia C., Trichopoulos D. (2003). Adherence to a Mediterranean diet and survival in a Greek population. N. Engl. J. Med..

[14] Searle S.D., Mitnitski A., Gahbauer E.A., Gill T.M., Rockwood K. (2008). A standard procedure for creating a frailty index. BMC Geriatr..

[15] Ware J., Kosinski M., Keller S.D. (1996). A 12-Item Short-Form Health Survey: Construction of scales and preliminary tests of reliability and validity. Med. Care.

[16] Irwin M., Artin K.H., Oxman M.N. (1999). Screening for depression in the older adult: Criterion validity of the 10-item Center for Epidemiological Studies Depression Scale (CES-D). Arch. Intern. Med..

[17] Folstein M.F., Folstein S.E., McHugh P.R. (1975). “Mini-mental state”. A practical method for grading the cognitive state of patients for the clinician. J. Psychiatr. Res..

[18] Bartali B., Frongillo E.A., Stipanuk M.H., Bandinelli S., Salvini S., Palli D., Morais J.A., Volpato S., Guralnik J.M., Ferrucci L. (2012). Protein intake and muscle strength in older persons: Does inflammation matter?. J. Am. Geriatr. Soc..

[19] Bartali B., Frongillo E.A., Bandinelli S., Lauretani F., Semba R.D., Fried L.P., Ferrucci L. (2006). Low nutrient intake is an essential component of frailty in older persons. J. Gerontol. A Biol. Sci. Med. Sci..

[20] Beasley J.M., LaCroix A.Z., Neuhouser M.L., Huang Y., Tinker L., Woods N., Michael Y., Curb J.D., Prentice R.L. (2010). Protein intake and incident frailty in the Women’s Health Initiative observational study. J. Am. Geriatr. Soc..

[21] Coelho-Junior H.J., Calvani R., Picca A., Tosato M., Landi F., Marzetti E. (2022). Protein Intake and Frailty in Older Adults: A Systematic Review and Meta-Analysis of Observational Studies. Nutrients.

[22] Kaimoto K., Yamashita M., Suzuki T., Makizako H., Koriyama C., Kubozono T., Takenaka T., Ohishi M., Kanouchi H., The Tarumizu Study Diet G. (2021). Association of Protein and Magnesium Intake with Prevalence of Prefrailty and Frailty in Community-Dwelling Older Japanese Women. J. Nutr. Sci. Vitaminol..

[23] Shikany J.M., Barrett-Connor E., Ensrud K.E., Cawthon P.M., Lewis C.E., Dam T.T., Shannon J., Redden D.T., Osteoporotic Fractures in Men Research Group (2014). Macronutrients, diet quality, and frailty in older men. J. Gerontol. A Biol. Sci. Med. Sci..

[24] Berner L.A., Becker G., Wise M., Doi J. (2013). Characterization of dietary protein among older adults in the United States: Amount, animal sources, and meal patterns. J. Acad. Nutr. Diet..

[25] Keller B.O., Wu B.T., Li S.S., Monga V., Innis S.M. (2013). Hypaphorine is present in human milk in association with consumption of legumes. J. Agric. Food Chem..

[26] Tanaka T., Talegawkar S.A., Jin Y., Candia J., Tian Q., Moaddel R., Simonsick E.M., Ferrucci L. (2022). Metabolomic Profile of Different Dietary Patterns and Their Association with Frailty Index in Community-Dwelling Older Men and Women. Nutrients.

[27] Playdon M.C., Moore S.C., Derkach A., Reedy J., Subar A.F., Sampson J.N., Albanes D., Gu F., Kontto J., Lassale C. (2017). Identifying biomarkers of dietary patterns by using metabolomics. Am. J. Clin. Nutr..

[28] Kim H., Appel L.J., Lichtenstein A.H., Wong K.E., Chatterjee N., Rhee E.P., Rebholz C.M. (2023). Metabolomic Profiles Associated With Blood Pressure Reduction in Response to the DASH and DASH-Sodium Dietary Interventions. Hypertension.

[29] Kim H., Lichtenstein A.H., White K., Wong K.E., Miller E.R., Coresh J., Appel L.J., Rebholz C.M. (2022). Plasma Metabolites Associated with a Protein-Rich Dietary Pattern: Results from the OmniHeart Trial. Mol. Nutr. Food Res..

[30] Sun H., Cai W., Wang X., Liu Y., Hou B., Zhu X., Qiu L. (2017). Vaccaria hypaphorine alleviates lipopolysaccharide-induced inflammation via inactivation of NFkappaB and ERK pathways in Raw 264.7 cells. BMC Complement. Altern. Med..

[31] Ding Y.H., Miao R.X., Zhang Q. (2021). Hypaphorine exerts anti-inflammatory effects in sepsis induced acute lung injury via modulating DUSP1/p38/JNK pathway. Kaohsiung J. Med. Sci..

[32] Bhave V.M., Ament Z., Patki A., Gao Y., Kijpaisalratana N., Guo B., Chaudhary N.S., Guarniz A.G., Gerszten R., Correa A. (2023). Plasma Metabolites Link Dietary Patterns to Stroke Risk. Ann. Neurol..

[33] Peron G., Merono T., Gargari G., Hidalgo-Liberona N., Minarro A., Lozano E.V., Castellano-Escuder P., Gonzalez-Dominguez R., Del Bo C., Bernardi S. (2022). A Polyphenol-Rich Diet Increases the Gut Microbiota Metabolite Indole 3-Propionic Acid in Older Adults with Preserved Kidney Function. Mol. Nutr. Food Res..

[34] Karbownik M., Stasiak M., Zygmunt A., Zasada K., Lewinski A. (2006). Protective effects of melatonin and indole-3-propionic acid against lipid peroxidation, caused by potassium bromate in the rat kidney. Cell Biochem. Funct..

[35] Bendheim P.E., Poeggeler B., Neria E., Ziv V., Pappolla M.A., Chain D.G. (2002). Development of indole-3-propionic acid (OXIGON) for Alzheimer’s disease. J. Mol. Neurosci..

[36] Aoki R., Aoki-Yoshida A., Suzuki C., Takayama Y. (2018). Indole-3-Pyruvic Acid, an Aryl Hydrocarbon Receptor Activator, Suppresses Experimental Colitis in Mice. J. Immunol..

[37] Xu X., Sun S., Liang L., Lou C., He Q., Ran M., Zhang L., Zhang J., Yan C., Yuan H. (2021). Role of the Aryl Hydrocarbon Receptor and Gut Microbiota-Derived Metabolites Indole-3-Acetic Acid in Sulforaphane Alleviates Hepatic Steatosis in Mice. Front Nutr..

[38] de Mello V.D., Paananen J., Lindstrom J., Lankinen M.A., Shi L., Kuusisto J., Pihlajamaki J., Auriola S., Lehtonen M., Rolandsson O. (2017). Indolepropionic acid and novel lipid metabolites are associated with a lower risk of type 2 diabetes in the Finnish Diabetes Prevention Study. Sci. Rep..

[39] Cason C.A., Dolan K.T., Sharma G., Tao M., Kulkarni R., Helenowski I.B., Doane B.M., Avram M.J., McDermott M.M., Chang E.B. (2018). Plasma microbiome-modulated indole- and phenyl-derived metabolites associate with advanced atherosclerosis and postoperative outcomes. J. Vasc. Surg..

[40] Rebholz C.M., Lichtenstein A.H., Zheng Z., Appel L.J., Coresh J. (2018). Serum untargeted metabolomic profile of the Dietary Approaches to Stop Hypertension (DASH) dietary pattern. Am. J. Clin. Nutr..

[41] Ramirez-Velez R., Martinez-Velilla N., Correa-Rodriguez M., Saez de Asteasu M.L., Zambom-Ferraresi F., Palomino-Echeverria S., Garcia-Hermoso A., Izquierdo M. (2022). Lipidomic signatures from physically frail and robust older adults at hospital admission. Geroscience.

